# Time Intervals in Sequence Sampling, Not Data Modifications, Have a Major Impact on Estimates of HIV Escape Rates

**DOI:** 10.3390/v10030099

**Published:** 2018-02-27

**Authors:** Vitaly V. Ganusov

**Affiliations:** 1 Department of Microbiology, University of Tennessee, Knoxville, TN 37996, USA; vitaly.ganusov@gmail.com; 2 Department of Mathematics, University of Tennessee, Knoxville, TN 37996, USA

**Keywords:** human immunodeficiency virus (HIV), cytotoxic T lymphocyte (CTL) response, viral escape, mathematical models

## Abstract

The ability of human immunodeficiency virus (HIV) to avoid recognition by humoral and cellular immunity (viral escape) is well-documented, but the strength of the immune response needed to cause such a viral escape remains poorly quantified. Several previous studies observed a more rapid escape of HIV from CD8 T cell responses in the acute phase of infection compared to chronic infection. The rate of HIV escape was estimated with the help of simple mathematical models, and results were interpreted to suggest that CD8 T cell responses causing escape in acute HIV infection may be more efficient at killing virus-infected cells than responses that cause escape in chronic infection, or alternatively, that early escapes occur in epitopes mutations in which there is minimal fitness cost to the virus. However, these conclusions were challenged on several grounds, including linkage and interference of multiple escape mutations due to a low population size and because of potential issues associated with modifying the data to estimate escape rates. Here we use a sampling method which does not require data modification to show that previous results on the decline of the viral escape rate with time since infection remain unchanged. However, using this method we also show that estimates of the escape rate are highly sensitive to the time interval between measurements, with longer intervals biasing estimates of the escape rate downwards. Our results thus suggest that data modifications for early and late escapes were not the primary reason for the observed decline in the escape rate with time since infection. However, longer sampling periods for escapes in chronic infection strongly influence estimates of the escape rate. More frequent sampling of viral sequences in chronic infection may improve our understanding of factors influencing the rate of HIV escape from CD8 T cell responses.

## 1. Introduction

Due to the absence of a proof-reading capability, human immunodeficiency virus (HIV)-1 (HIV hereafter) has a relatively high average mutation rate of 2–4$\times 10^{-5}$ per bp per replication cycle [1,2,3,4,5]; however, even higher mutation rates have been suggested [5,6]. Given a relatively short genome (∼$10^{4}$ bp), mutant viruses arise only in 20–40% of replication cycles. Even with such perhaps surprising fidelity given a short virus replication cycle time (∼1–2 days, [7]) and large population size (∼10${}^{7}$–10${}^{8}$ of virus-infected cells [8,9]), HIV is able to generate multiple variants during the course of infection. Some of these variants will be unrecognized by the host’s immune responses, resulting in escape, which may prevent viral control. Indeed, the ability of HIV to escape from cellular (CD8 T cell) and humoral (antibody) responses is often defined as the major obstacle preventing the development of efficient vaccines [10,11,12,13]. One of the early mathematical models of why HIV-infected individuals progress to acquired immunodeficiency syndrome (AIDS) also involves viral escape from immunity [14,15].

While HIV clearly escapes from both cellular and humoral immune responses, frequent sampling of the escape dynamics suggested that escape from virus-specific CD8 T cell responses occurs earlier than from antibody-mediated responses [16,17,18,19]. Escape from CD8 T cell responses has been accurately mapped for several HIV-infected individuals from the earliest stages of infection [16,19,20]. Specifically, these studies tracked changes in the HIV genome from the virus sequence founding the infection (transmitted/founder virus) and mapped CD8 T cell response specific to the whole viral proteome. Genetic changes in regions recognized by CD8 T cells was treated as evidence of T cell recognition of the virus, and thus the speed at which the founder viral sequence was lost was indicative of T cell response strength. Multiple studies developed mathematical models to estimate the rate at which founding wild-type virus was lost from the population (and consequently, the escape variants accumulated in the population); this escape rate was then interpreted as the rate at which epitope-specific CD8 T cells eliminate HIV-infected cells [21,22,23,24,25,26,27]. Estimation of HIV escape rates was deemed complicated due to the sparse nature of the sequence data coming from the relatively infrequent sampling of virus populations and a low number of viral sequences obtained at a given time point in most studies. In particular, many of the data on escape measuring frequency of the wild-type (or escape) variant include measurements of only either 100% or 0% in two sequential time points (or in some cases, with one intermediate frequency of the wild-type virus between the two extreme values of 100% and 0%). To estimate HIV escape rates in such data, the data were modified to include some presence of the escape variant or the wild-type virus [21,22]. For example, if out of 10 sequences of a specific epitope region 10 sequences were of the wild-type virus, it was proposed to add 1 extra sequence with a mutated epitope, resulting in the frequency of the wild-type $f_{w}=10/11=91\%$ instead of the observed 100%. Similarly, if 5 out 5 sequences at the subsequent time point were mutated, adding 1 wild-type sequence resulted in the wild-type variant frequency of $f_{w}=1/6=17\%$ instead of 0%. Such modifications were proposed to provide minimal escape rates [21,25]. It remained unknown whether such data modifications were important for making general observations regarding HIV escape rates; previous studies utilizing such data modifications suggested that the rate of HIV escape declines with time since infection, implying a potential weakening of the immune responses or a simple consequence of the virus escaping in the least costly positions [25,28]. More recent studies have highlighted that the proposed data modification may have had a strong impact on estimated escape rates, thus questioning the validity of previous conclusions [29,30]. Here we extend previous analyses by applying a novel method of estimating rates of viral escape which does not involve modification of the data, using previously published data. The method uses sampling of the sequence data using the beta distribution, which serves as a continuous approximation of the binomial distribution. With this sampling method, we show that at least for three HIV-infected patients, previous data modifications were not responsible for the predicted decline of the escape rate with time since infection. However, the sampling method remains highly sensitive to the time frequency of data sampling—the method estimates slower escapes for less-frequently-sampled data. This analysis suggests that better understanding of the mechanisms behind HIV escape from CD8 T cell responses is not likely to come from improved methods of sequence data analysis, but from better data with improved time resolution and depth of sequencing.

## 2. Materials and Methods

**Data**. Experimental data used in this paper are from previous publications [16,25]. In short, individuals with recent HIV-1 infection were recruited into the study. Patients donated blood at regular time intervals, and viral RNA sequences were obtained using single genome amplification (SGA) techniques. Three patients from the Center for HIV/AIDS Vaccine Immunology (CHAVI) were analyzed: CH40, CH77, and CH58. These patients were infected with a single transmitted/founder virus, and changes in the viral genome were mapped to the HIV-specific cytotoxic T lymphocyte (CTL) responses. In viral sequences, there were also changes that had signatures of viral escape from CTL responses, but no CTL responses specific to proteins in these specific regions have been detected [16]. We re-analyzed viral sequence data for all detected escapes and restricted some analyses to only escapes from detected CTL responses. Subjects are controlled in accordance with the tenets of the Declaration of Helsinki.

**Model**. We used a previously suggested mathematical model of viral escape from a single CTL response [21,22,23,25]. The model was fit to experimental data using likelihood method based on binomial distribution (Equation (2) [31]).

**Statistics**. When fitting mathematical models to experimental data, it is important to estimate confidence intervals for the model parameters. One widely-used approach is bootstrap, in which either experimental data or residuals (difference between model prediction and the data) are sampled with replacement [32]. Two types of bootstraps exit: non-parametric and parametric bootstrap. In nonparametric bootstrap, data are sampled with replacement into a bootstrap sample and then the model is fitted to the sample dataset. For example, one could resample with replacement residuals from the model fit of the data, add resampled residuals to the model predictions to generate a sampled dataset, and fit the model to these sampled data. In contrast, in parameteric bootstrap, one assumes a particular distribution underlying the data and generates samples using this distribution. For example, residuals resulting from a given model fit may follow a normal distribution with zero mean and variance $\sigma^{2}$. Therefore, using normal distribution $N(0,\sigma^{2})$, one could generate another bootstrap sample by adding randomly generated residuals to predictions of the model best fit [32]. In our analysis, we use a version of the parametric bootstrap whereby we generate samples of data assuming that escape variant frequency follows a beta distribution with *m* mutant and *w* wild-type sequences. A beta distribution is a continuous approximation of the binomial distribution, and the beta distribution has been used to calculate confidence intervals for proportions [33].

**Programming**. Model fits of the data were done in Mathematica 11 using a routine FindMinimum. All codes are available from the author upon request. An example of a Mathematica notebook illustrating the strategy of the sampling method to estimate escape rates is available as a supplement to this paper.

## 3. Results

### 3.1. A Sampling Method to Estimate Escape Rates without Data Modification

Several previous studies have shown that the kinetics of viral escape from a single CD8 T cell response can be described by the logistic equation

(1) $$f\left(t\right)=\frac{f_{0}}{f_{0}+\left(1-f_{0}\right)e^{-\varepsilon t}},$$

where $f_{0}$ and f(t) are the frequency of the escape variant at time t=0 and time *t*, respectively, and ε is the escape rate [21,22,23,25]. When the escape variant is generated by mutation from the wild type, the predicted dynamics of the frequency of the escape variant is approximately given by the logistic equation [31]. Because the generation of escape variants is a stochastic process, this simple model can be interpreted as describing virus dynamics when an escape variant reached some sufficient frequency when the importance of genetic drift on virus dynamics becomes negligible. The time at which the wild-type or mutant variant reaches 50% is called $t_{50}=\frac{1}{\varepsilon }\log\left(\frac{1-f_{0}}{f_{0}}\right)$ [25]. The use of a deterministic model that ignores the stochasticity of virus dynamics could in part be justified by the large population size of HIV during acute and early HIV infection, reaching $10^{7}-10^{9}$ infected CD4 T cells [8,9] and high virus mutation rate on the order of ∼$10^{-5}$ [1,2,3,4,5,6].

Data on viral escape are generally given as the percent of escape variant in the viral population at different time points with a number of viral sequences obtained. For example, at time 10 days post symptoms, 15 viral sequences were obtained, and 3 of these contained mutated epitopes, making the frequency of the escape variant 3/15 or 20%. To estimate escape rate it, is advised to use a maximum likelihood approach [31] in which the binomial distribution-based likelihood of the model given experimental data is given by

(2) $$L∼\prod_{i=1}^{n}f{\left(t_{i}\right)}^{m_{i}}{\left(1-f\left(t_{i}\right)\right)}^{w_{i}},$$

where $m_{i}$ and $w_{i}$ are the number of escape mutant and wild-type sequences in the ith sample taken at time $t_{i}$ and there are *n* time points. Note that we ignored constant terms for the binomial distribution since these do not matter during likelihood maximization. Parameters of the model including the escape rate ε and initial variant frequency $f_{0}$ are then found by maximizing likelihood [31]. As discussed in the introduction, the maximization of the likelihood function Equation (2) fails for finite values of the escape rate if the data do not have measurements in which both wild-type and mutant sequences are observed. In some cases when there is only one time point at which both wild-type and mutant viruses are detected and for all other time points only one variant is observed, it is possible to estimate the escape rate without data modification [34].

To obtain an estimate of the escape rate for the data when there are only wild-type or only mutant variants detected at a given time point, we propose to use a parameteric bootstrap approach. In this approach, we do not fit the basic mathematical model (Equation (1)) to the actual data. Instead, we generate pseudorandom samples of the data which are found using a beta distribution for every sample in every time point [33]. Specifically, for the $N_{i}=w_{i}+m_{i}$ sampled sequences at time $t_{i}$, where $m_{i}$ is the number of escape variant sequences and $w_{i}$ is the number of wild-type sequences, the resampled frequency of the escape variant $x_{i}$ is given by the beta distribution $Beta(m_{i}+1/2,w_{i}+1/2)$ [33]. This procedure is repeated for all *n* time points, resulting in a bootstrap sample. In our analyses, we used the routine Random in Mathematica. Due to randomness, the resulting bootstrapped sample will have both wild-type and escape variants present at multiple time points. The model can then be fitted to these bootstrapped data using the likelihood approach according to Equation (2). Repeating this sampling procedure a given number of times (e.g., $10^{3}$ simulations), a distribution of escape rates, initial mutant frequency, and the time to 50% of the escape variants can be generated. The mean (or median) of these distributions and 95% interval of the distribution can be then used to characterize the escape rate ε and the time to 50% escape variant $t_{50}$.

**Figure 1:**
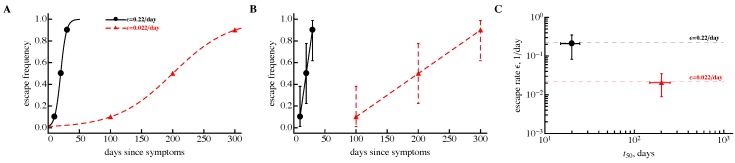
A sampling method accurately recovers virus escape rate for data when both wild-type and escape variant are detected at multiple sequential time points. We simulated viral escape using a logistic equation with two escape rates: ε=0.22/day (bullets) and ε=0.022/day (triangles). Three data points at which the escape variant was present at 10%, 50%, and 90% out of N=10 sequences were generated (panel **A**). These simulated data were fitted by the simple logistic model (Equation (1)) using a likelihood approach, leading to expected escape rates (lines in panel A). These data were then resampled using the beta distribution (panel **B**, black solid line is for ε=0.22/day and red dashed line is for ε=0.022/day) and fitted by the same model using the likelihood method. Resulting averages and their 95% confidence intervals match well with the numbers estimated using actual data (panel **C**). Dashed lines in panel (**C**) show estimates of the escape rates found in panel (**A**), and 95% confidence intervals in panel (**B**) are predicted using Jeffrey’s intervals [33].

While the sampling method was specifically designed to estimate escape rates from the data which cannot be fit by simple mathematical models (e.g., as in Equation (1), it was important to confirm that for well-sampled data the method was able to recover the escape rate that would be found by fitting the models directly to such data. As a proof of principle, we simulated viral escape using the logistic model (Equation (1)) and assumed that it was possible to detect both wild-type and escape variant viruses at three sequential time points (Figure 1). Obviously, the logistic model can well fit these data and recover a correct escape rate value (Figure 1A) without the need for additional methods. Importantly, however, sampling these data using the beta distribution and estimating the escape rate for these bootstrap samples allowed for a relatively accurate estimate of the fast and slow escape rates (Figure 1C). Additional simulations with more randomly chosen escape rates and samplings confirmed that the method of sampling recovered the same escape as the model fitted directly to the escape data. The results of these analyses were similar if we used data in which both wild-type and escape variants were present at two sequential time points (results not shown). In our simulations, sampling using beta distribution never yielded a 0% or 100% for the wild-type virus, and thus always allowed us to find a finite value for the average escape rate.

### 3.2. The Rate of HIV Escape Declines with Time since Infection

**Figure 2:**
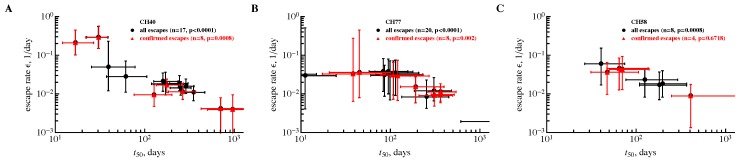
A sampling method which does not involve data modification still predicts a decline in the estimated escape rate with the time since infection. We used a novel approach of data sampling and estimated the rate of human immunodeficiency virus (HIV) escape from CD8 T cell responses for three Center for HIV/AIDS Vaccine Immunology (CHAVI) patients using previously published data [16,25]. Rates (ε) and time to 50% of the escape variant ($t_{50}$) and associated errors are shown for three different patients in different panels (panel **A**: CH40, panel **B**: CH77, panel **C**: CH58); bullets indicate all escapes (in black) and triangles are for confirmed escapes (in red) [16]. Confirmed escapes were defined as fixed changes in HIV genome with a clear signature of viral escape with detected epitope-specific T cell response. Other escapes had a well-defined signature of escape but no epitope-specific T cell response was detected [16]. Resulting *p* values from the Spearman rank correlation test are indicated on individual panels. Panel (**B**) does not show an estimate for one escape due to very low escape rate ε and long $t_{50}$.

**Figure 3:**
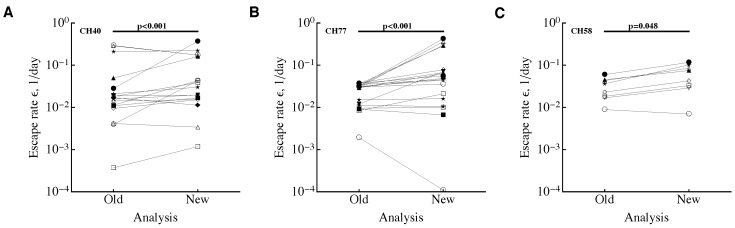
A sampling method provides higher estimates of the escape rates. We plot estimates of HIV escape rates in three patients (panel **A**: CH40, panel **B**: CH77, panel **C**: CH58) as was found a previous study [25] employing data modifications (“Old” analysis) and estimates found using sampling method (“New” analysis). Different markers on plots indicate escape rate estimates for different escapes in our previous analysis [25]. Comparison of the escape rates in two analyses was done using Wilcoxon signed rank test and *p* values from these tests are indicated on individual panels.

Previous work from several groups suggested that during HIV infection the rate of HIV escapes from T cell responses declines over time [25,28]. However, this conclusion was challenged on multiple levels, including the need for modification of the data to provide minimal estimates of the escape rate [29]. Indeed, it was possible that for many late escapes, previous studies provided minimal estimates of the escape rate and thus the observed pattern of the escape rate decline with time since infection was an artifact of data modification [26,29,30]. To address this specific point, we re-analyzed previously published data on HIV escape [16,25]. Specifically, we used data for all documented escapes in three patients from the Center for HIV/AIDS Vaccine Immunology (CHAVI) as described in previous publications, and limited these data to the first 800 days post-infection. This was done because of the bias towards lower estimates the sampling method introduces in datasets with longer sampling times (see Discussion). We then resampled data for all escapes, fitted the simple logistic model (Equation (1)) to these data, and calculated the average escape rate (ε) and time of escape ($t_{50}$). This reanalysis revealed a consistent and statistically significant decline in the estimated rate of HIV escape from CD8 T cell responses with the time since infection (Figure 2). This was true for all documented escapes (i.e., escapes which had the pattern of viral escape from T cell responses but may or may not have had measurable T cell response against the wild-type epitope) in two out of three patients with escapes with detected T cell responses against the wild-type epitope. The poor correlation in the latter case in patient CH58 is likely due to a small number of confirmed escapes (n=4). Interestingly, there was a statistically significant correlation between mean escape rates obtained from parametric sampling of the data and previous estimates found with data modifications; however, the novel sampling method consistently provided higher estimates of the escape rate than previously reported (Figure 3) [25]. This is consistent with a recent report that used deep sequencing in patients in acute HIV infection and found higher estimates of the HIV escape rates from CD8 T cell responses [19]. Taken together, our analysis suggests that data modification was not the reason for the detected decline in the escape rate with time since infection [25].

### 3.3. Time Frequency of Sampling Biases Estimates of the Escape Rate

**Figure 4:**
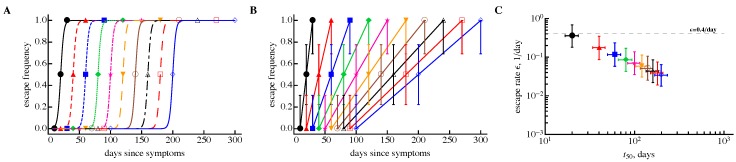
Time interval between measurements has a strong bias on the estimate of the escape rate and leads to a correlation between the escape rate (ε) and time of escape ($t_{50}$). We generated artificial data in which viral sequences were measured in three time points, resulting in 0%, 50%, and 100% of the escape variant with N=10 sequences per time point (panel **A**). In all cases, escape can be described by the logistic equation (Equation (1)) assuming a delayed immune response and escape rate ε=0.4/day. We resampled these simulated “data” using a beta distribution (panel **B**, different lines indicate different escapes as in panel **A**) and estimated the average escape rate (panel **C**). We show the prediction of the logistic curve obtained using ε=0.4/day (panel **A**), sampled data (panel **B**), and estimates of the escape rate obtained using parametric bootstrap (panel **C**). The horizontal dashed line in (panel **C**) denotes the theoretical escape rate of 0.4/day. The 95% confidence intervals in (panel **B**) are predicted using Jeffrey’s intervals [33], and in (panel **C**) CIs were calculated from bootstrapped samples.

Our results so far suggest that data modifications were not likely responsible for the observed decline in the estimated escape rate with time since infection. Therefore, it is important to understand whether other features in the data could be responsible for this declining trend. Previous studies have highlighted that the frequency of sampling in experimental data have changed over time, starting from weekly sampling and changing to monthly/bi-monthly sampling [16]. Such a pattern may naturally lead to underestimates of escape rates in part due to clonal interference between different escape variants [26,29,30]. Therefore, we sought to determine if the sampling method was capable of accurately recovering the escape rate independently of the sampling time period. We simulated viral escape with the same escape rate but assumed different initial frequency for the virus and different time intervals between measurements. This allowed wild-type and escape variant to be present in the data only at one time point (Figure 4A). The resulting “data” looked somewhat similar to those observed during HIV infection (Figure 4B). By fitting the logistic model to bootstrapped samples, we found that the sampling period had a dramatic impact on the estimated escape rate (Figure 4C). In an extreme case, the method underestimated the escape rate 10-fold! Thus, sampling frequency (i.e., weekly, monthly, bi-monthly, etc.) has a dramatic impact on the estimate of the escape rate even in the absence of data modification and when one uses a sampling method. This result has two consequences. First, in order to understand the kinetics of HIV escape from T cell responses, more frequent sampling during chronic HIV infection will be needed. Second, previous results on the independence of the escape rate on the magnitude of the T cell response [25] and the decline of the escape rate with the time since infection need to be re-evaluated using additional, perhaps better sampled data.

## 4. Discussion

Several lines of evidence suggest that CD8 T cell responses play an important role in control of HIV replication [16,35,36]. Escape of HIV from T cell responses is one type of indirect evidence in support of this hypothesis. Accurate estimates of the rate at which CD8 T cells are eliminating the virus-infected cells (or more generally, suppressing virus replication) and comparison of these rates with other kinetic parameters of HIV replication in vivo may be useful in testing this hypothesis further [22]. The rate at which HIV escapes from CD8 T cell responses may be one metric indicating the strength of the HIV-specific CD8 T cell response. However, multiple studies have now shown that estimating the rate of HIV escape using current data is complicated.

We have proposed a new method of sampling sequence data to estimate escape rates in situations when direct fitting of simple deterministic mathematical models to the data fails. An alternative approach to estimating escape rates from sparsely sampled data would be to use more mechanistic stochastic models of HIV dynamics and evolution; for example, models that take into account virus effective population size, as well as the rate of HIV mutation and recombination [26]. However, it is unclear how well such complex models with multiple parameters represent HIV dynamics in vivo, and as such, biases in parameter estimates associated with such models are not known. Combining the use of such stochastic models with the method of data sampling using the beta distribution may allow quantification of potential biases in parameter estimates in the models and to further understand mechanisms behind HIV escape from CD8 T cell responses.

Using the sampling method, we have shown that the observed phenomenon of a decline of the rate of HIV escape with time since infection is not the result of modification of data to accommodate fits of simple models of viral escape. However, the method suffers from a great sensitivity to the time frequency of sampling in the data, since increasing the time intervals between measurements naturally decreases the estimated escape rate. Furthermore, the inclusion of more data at later time points in which wild-type virus is nearly lost will also bias average estimates found by sampling downwards, which further limits the applicability of this method for the estimation of escape rates. Specifically, if for the first escape in Figure 1A we added more measurements at later time points (e.g., at day 100 or 300, when the escape variant is likely to be at 100% frequency), sampling would have provided much lower estimates of the escape rate than the ε=0.22/day found by the model fitting to the original, not resampled data (results not shown). The sampling method also requires more computational power, as at least $10^{3}$ samples are generally required to accurately estimate confidence intervals [32]. While fitting a simple logistic model to escape data is generally fast, fitting more complicated models assuming, for example, sequential or concurrent escape, can be time-consuming [34]. Finally, the method relies on the approximation of the binomial distribution by the beta distribution, which may fail in some circumstances [33].

Overall, our results so far suggest that the current state of data on HIV escape does not allow us to make solid inferences on the escape rate without making strong assumptions either about the data or about the underlying dynamics of the virus. It appears that the way forward is to generate novel data in which patient sampling occurs at regular intervals over the course of a few months of infection, perhaps with the use of novel techniques such as deep sequencing. Consistent temporal sampling rather than deep sampling is likely to be more beneficial for determining if the rate of HIV escape from T cell responses declines with time since infection [31].

## Supplementary Materials

The following are available online at http://www.mdpi.com/1999-4915/10/3/99/s1.
